# 1453. Whole Genome Sequencing: A Useful Tool in a VRE Outbreak Among Immunocompromised Pediatric Patients

**DOI:** 10.1093/ofid/ofad500.1290

**Published:** 2023-11-27

**Authors:** Catherine R Murphy, Sheetal Mahulkar, Gladys Griffin, Cameron Griffin, Andrea L Ankrum, William R Otto, Lara A Danziger-Isakov, David Haslam, Felicia Scaggs-Huang

**Affiliations:** Cincinnati Children's Hospital Medical Center, Cincinnati, Ohio; Cincinnati Children’s Hospital and Medical Center, Cincinnati, Ohio; CCHMC, Cincinnati, Ohio; Cincinnati Children's Hospital Medical Center, Cincinnati, Ohio; Cincinnati Children's Hospital Medical Center, Cincinnati, Ohio; Cincinnati Children's Hospital Medical Center, Cincinnati, Ohio; Cincinnati Children's Hospital, Cincinnati, Ohio; Cincinnati Children's Hospital Medical Center, Cincinnati, Ohio; Cincinnati Children's Hospital Medical Center, Cincinnati, Ohio

## Abstract

**Background:**

Vancomycin-resistant enterococcus (VRE) colonization and infection is an increasing threat in pediatric oncology and bone marrow transplant (BMT) patients. We identified a cluster of 13 hospitalized patients who acquired VRE (*E. faecium*) over a six-month period at a quaternary children’s hospital.

**Methods:**

An outbreak response was coordinated among Infection Prevention & Control (IP&C) and leadership of affected units. We defined a case as a hospitalized patient with new VRE colonization or infection beginning in April 2022. We reviewed electronic medical records of case patients and IP&C practice adherence on their service units, including biweekly VRE stool screening on BMT. Whole genome sequencing (WGS) was performed on all cluster isolates and pairwise single nucleotide polymorphisms (SNPs) were calculated. Three additional VRE isolates obtained at our institution within a 1-year period before the cluster were processed for comparison.

**Results:**

From April 14 to October 21, 2022, 7 VRE healthcare-associated infections and 6 colonization events were identified on BMT and Oncology. Healthcare worker (HCW) hand hygiene (HH) adherence based on World Health Organization’s 5 moments was >95%, but supplemental observations during the outbreak period noted HH adherence of 70%. Personal protective equipment (PPE) adherence based on Centers for Disease Control and institutional requirements was 70-80% depending on the observed element. On BMT, VRE screening was completed on 33% of patients during admission; screening is not performed on Oncology. Although there was temporo-spatial overlap in some cases, no clear epidemiologic links were found. However, phylogenetic analysis revealed 11/13 (85%) isolates were closely-related (Table 1, Figure 1). Improvement opportunities were shared with BMT and Oncology and no further VRE cases were identified.

**Figure:**
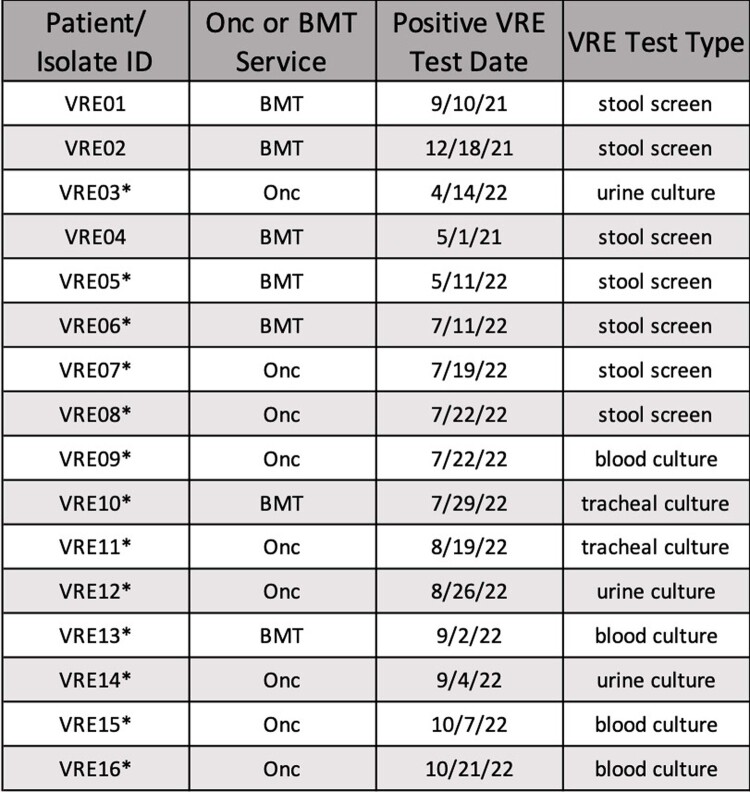

All VRE isolates sequenced and analyzed. Thirteen cluster isolates (VRE03 and VRE05-16) are starred (*). Three comparison isolates (VRE01, VRE02 and VRE04) are not starred. Abbreviations: Onc = Oncology, POA = present on arrival; HAC = hospital-acquired colonization; HAI = hospital-acquired infection.

**Figure:**
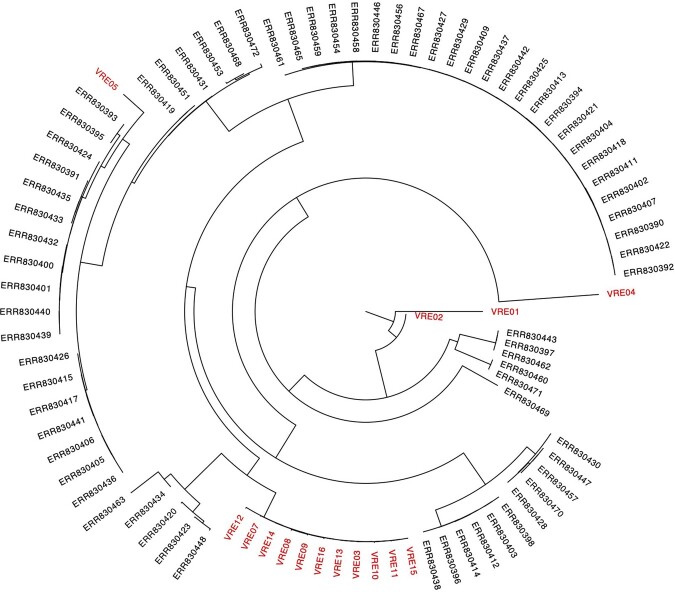

Phylogenetic analysis of 16 VRE isolates (13 cluster and 3 comparison) obtained in our institution (highlighted in red) compared with multiple reference VRE isolates. Eleven of thirteen cluster isolates (VRE03 and VRE07-VRE16) were revealed to be closely-related. Of note, VRE06 was so genetically dissimilar from the other cluster and comparison isolates, it was removed from this figure.

**Conclusion:**

We identified a likely nosocomial transmission of VRE among pediatric Oncology and BMT patients. Although no clear epidemiological links were found on extensive investigation, WGS revealed transmissions among patients occurred, reinforcing the importance of adherence to IP&C measures such as HH compliance, PPE utilization, and VRE screening for outbreak prevention in healthcare facilities.

**Disclosures:**

**William R. Otto, MD, MSCE**, Moderna: Grant/Research Support **Lara A. Danziger-Isakov, MD, MPH**, Aicuris: Contracted Clinical Research|Ansun Biopharma: Contracted Clinical Research|Astellas: Contracted Clinical Research|GSK: Advisor/Consultant|Merck: Advisor/Consultant|Merck: Contracted Clinical Research|Pfizer: Contracted Clinical Research|Roche Diagnostics: Advisor/Consultant|Takeda: Advisor/Consultant|Takeda: Contracted Clinical Research

